# Phytochemical Characterization of *Pouteria guianensis*: Optimized Flavonoid Extraction and HPLC Method Validation

**DOI:** 10.1021/acsomega.5c03580

**Published:** 2025-07-14

**Authors:** Rafael Christian de Matos, Ana Flávia Alvarenga Bitencourt, Alexsandro Davi Mantovani de Oliveira, Carolina Paula de Souza Moreira, Renes Resende Machado, Marina Scopel

**Affiliations:** † Faculdade de Farmácia, 605143 Universidade Federal de Minas Gerais, Avenida Antônio Carlos 6627, Campus Pampulha, 31.270-901 Belo Horizonte, MG, Brazil; ‡ Centro Especializado Em Plantas Aromáticas, Medicinais e Tóxicas - CEPLAMT-Museu de História Natural e Jardim Botânico da Universidade Federal de Minas Gerais, Rua Gustavo da Silveira 1035, Horto, 31.080-010 Belo Horizonte, MG, Brazil; § Fundação Ezequiel Dias, Rua Conde Pereira Carneiro 80, Gameleira, 30.510-010 Belo Horizonte, MG, Brazil

## Abstract

Plant biodiversity of Brazil offers significant potential for the development of phytotherapeutics, highlighting the *Pouteria* spp. genus. Despite traditional use and ethnopharmacological support, *Pouteria* species remain underexplored in scientific literature. This study aims to explore the flavonoids class, by optimizing flavonoid extraction from *Pouteria guianensis* (Aubl.) leaves through response surface methodology and by developing and validating a high-performance liquid chromatography (HPLC) method. Parameters reported in the literature as important for the extraction of this genus were optimized using parametric statistical analysis. Extraction optimization was achieved evaluating key parameters, including extraction time (5,10 and 15 min), drug-to-solvent ratio (1:5, 1:10 and 1:15), temperature (20, 30 and 40 °C), and the number of extraction cycles (1, 2 and 3). The developed HPLC method was validated using myricitrin as the primary analytical marker and quercetin was chosen as external standard for quantitative purposes. Temperature was found to play a pivotal role in metabolite extraction from the plant raw material and the optimized condition consisted of two extraction cycles, each lasting 8 min, at 21 °C, with a drug-to-solvent ratio of 1:8. The optimized *P. guianensis* extract (10 mg/mL) was analyzed by HPLC using methanol as the diluent, with quercetin (29.5 μg/mL) as an external marker. The myricitrin content in the plant was determined to be 2.96 mg/g (0.30%). This study provides the first detailed characterization of a chemical marker for the *P. guianensis* species using an optimized extraction process that can be useful for other species of the genus in the future.

## Graphical abstract

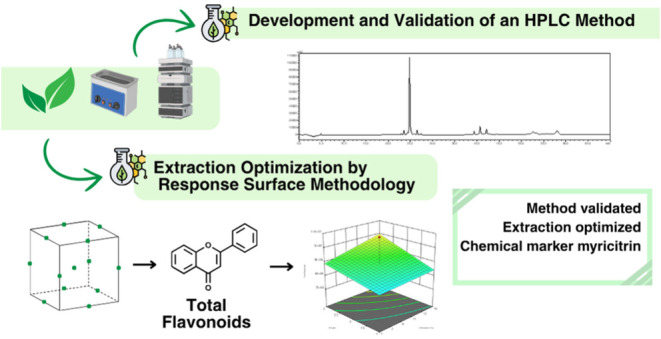

## 1 Introduction

The use of medicinal plants for palliative and therapeutic purposes dates back to the earliest periods of human history and remains practiced worldwide.^1^ Brazil hosts one of the largest and most diverse flora on the planet, offering substantial potential for the development and discovery of new herbal medicines; however, much of this biodiversity remains underexplored.^2^

The genus *Pouteria* spp. stands out as an example of an unexploited genus. Some of the species from this genus contain a rich array of phenolic compounds and have been associated with various ethnopharmacological uses, including analgesia, inflammation, malaria fever, and gastrointestinal disorders.^3–10^

Recent studies have reported the presence of alkaloids in *Pouteria* leaves—a rare class of compounds for the genus,^11^ indicating the need for further research into the special metabolites of this genus.

*Pouteria guianensis* (Aubl.) is a member of the genus *Pouteria* and, despite some botanical studies,^12^ it remains challenging to differentiate it from other morphologically and anatomically similar species.^9^ Additionally, the leaves of *Pouteria* spp. contain latex with known toxic properties,^13^ demanding the need for careful evaluation of their possible herbal preparations.^14–16^ This highlights the importance of defining the optimal extraction method to ensure safe use.

Previous analytical efforts on *Pouteria* species have primarily focused on untargeted metabolomics and general phytochemical screening, often lacking robust quantification methods. Furthermore, the only validated analytical method currently available within the genus pertains to the fruits of Pouteria macrophylla, making the present study the first to report a validated method specifically for *Pouteria* leaves.^17–19^

Moreover, while HPLC remains a cornerstone in the analysis of phenolic compounds due to its sensitivity, reproducibility, and cost-effectiveness, few studies have validated such methods specifically for *P. guianensis*, particularly with regard to method robustness, matrix effects, and selectivity.^20^ In this context, integrating extraction optimization with analytical method development becomes essential to ensure both the chemical integrity and safety of plant-derived preparations. Techniques such as response surface methodology (RSM), factorial design, and multivariate data analysis have been successfully applied to optimize extraction protocols in other plant matrices, yet they are underutilized in Pouteria leaves research.^21^

In summary, this study aims to optimize the extraction of an 80% hydroethanolic extract from *P. guianensis* leaves, select a chemical marker for the species, develop and validate an HPLC method, and propose chemical differentiations among the various *Pouteria* species.

## 2 Materials and Methods

### 2.1 Plant Material

Leaves from four species of *Pouteria* (Sapotaceae) were collected in Minas Gerais, Brazil (latitude*:* −19.9208, longitude: −43.9378, error: ± 17082) and deposited in the herbarium of the Federal University of Minas Gerais (BHCB). The species collected include *P. guianesis* Aubl. (BHCB210380), *Pouteria torta* (Mart.) Radlk. (BHCB210385), *Pouteria caimito* Radlk. (BCHCB210384), and *Pouteria gardneri* (Mart. & Miq.) Baehni. (BHCB210381). The leaves were dried in an air-circulating oven at a temperature of 40 °C with air circulation for 7 days, followed by grinding through a 355 μm mesh.

### 2.2 Chemicals

Quercetin (purity ≥ 95%), myricitrin (purity ≥ 99%), and 2-diphenyl-1-picrylhydrazyl (DPPH) were obtained from Sigma-Aldrich (St. Louis, MO). Methanol and acetonitrile (HPLC grade) were purchased from Merck (Buenos Aires, Argentina). All other reagents used in the study were of analytical grade.

### 2.3 Experimental Design

Four distinct variables, each at three different levels, were selected to optimize the extraction of phenolic compounds from *P. guianensis* leaves,^22–24^ as shown in Table 1. Ultrasound-assisted extraction was performed using 80% hydroethanolic solution as the solvent, and the experimental design followed a 2^4^ factorial, with two replicates and a single blocking system, executed in *Design-Expert* software (State-Ease, Design-Expert software, version 13.0.12.0).

**Table 1:** Extraction Variables and Three-Level Factorial Design

		levels		
codes	factor	-1	0	1
A	extraction time (min.)	5.00	10.00	15.00
B	number of cycles	1	2	3
C	temperature (°C)	20.00 (±2.00)	30.00 (±2.00)	40.00 (±2.00)
D	drug/solvent ratio (g/mL)	1: 5	1: 10	1: 15

Randomization of the experiments, performed by the software, resulted in 33 independent trials, which were subsequently analyzed by HPLC. Following each extraction, samples were diluted to 10 mg/mL with methanol, and the total area of all the chromatographic peaks corresponding to flavonoids in the ultraviolet spectrum was recorded as the dependent variable. Data were analyzed using parametric statistical methods, including multivariate linear regression, residual analysis, analysis of variance (ANOVA), and the generation of binary and ternary surface plots, according to the following equation

$$Y=\beta_{0}+\sum_{i=1}^{k}\beta_{i}x_{i+}\sum_{\begin{array}{l} i=1\\ i<j \end{array}}^{k=1}\sum_{j=2}^{k}\beta_{\mathrm{ij}}x_{j}+\sum_{i=2}^{k}\beta_{\mathrm{ii}}x_{\mathrm{ii}}^{2}$$

where β_0_, β*_i_*, β*_ii_*, and β*_ij_* are the regression coefficients; *x*_*i*_ and *x*_*j*_ are the coded levels of independent variables affecting the dependent response *Y*; *k* is the number of parameters.

### 2.4 Chromatographic Analysis

#### 2.4.1 Sample and Standard Preparation

For the quantitative analysis of *P. guianensis* extract by HPLC, the optimized extract was prepared at 10 mg/mL using methanol as the diluent. For quantification, a sample of quercetin (29.5 μg/mL) (external marker) was prepared in the same diluent on the day of analysis, to compare the analyte areas relative to the standard.

To enhance the chemical differentiation of the four *Pouteria* samples, a hydrolysis procedure described by Matos et al. (2024) was used to evaluate the flavonoid aglycones present in the extracts. This method involved refluxing 0.4 g of the powdered plant material with 1.18 M HCl in a hydro-methanol solution (50:50, v/v) at 90 °C for 205 min. After this period, the sample was filtered, and the remaining plant material was refluxed with an additional 10 mL of the acid solution for an additional 10 min, repeating this extraction cycle. The combined extracts were dried under reduced pressure using a rotary evaporator, resuspended in 10 mL of water, and extracted with ethyl acetate (3 × 10 mL). The combined organic fractions were washed with ultrapure water (2 × 50 mL) and filtered through 1 g of anhydrous sodium sulfate. The extracts were then concentrated under reduced pressure, resuspended in 5 mL methanol, and diluted with the same solvent at a 1:5 ratio. These solutions were filtered through a 0.45 μm PVDF membrane and analyzed by HPLC.

To confirm the identity of the main peak of *P. guianensis*, two independent experiments were conducted. First, a standard coinjection test was performed using the extract (myricitrin at 65.8 μg/mL). Second, a 50 ng/mL sample was analyzed using Triple Quad LC-ESI-MS/MS. Characteristic fragmentations for myricitrin were identified based on the MassBank public in-house database,^25^ with fragmentation patterns observed in positive mode (*m*/*z* 465 → *m*/*z* 319) and negative mode (*m*/*z* 463→ *m*/*z* 317, *m*/*z* 287, and *m*/*z* 271).

HPLC analyses were conducted by injecting 10 μL of each sample into a Shimadzu LC-20A Prominence chromatograph coupled with a photodiode array detector (Shimadzu SPD-20A). The separation was carried out at 40 °C using an NST C_18_ reversed-phase column (250 mm × 4.6 mm, 5 μm particle size). A gradient elution method was optimized for this study (data not shown), using aqueous 0.2% phosphoric acid as mobile phase A and 0.2% phosphoric acid in methanol as mobile phase B. The gradient program at a flow rate of 0.6 mL/min was as follows: 5–95% B (0–30 min), 95% B (30–60 min), 95–5% B (60–65 min), and 95–5% B (65–70 min). Chromatographic data were collected at 370 nm, and data acquisition was conducted using Shimadzu Lab Solutions software.

Mass spectrometric analysis was performed using an Agilent Eclipse Plus RR C_18_ column (2.1 mm × 50 mm, 1.8 μm particle size), with mobile phase consisting of (A) ultrapurified water and (B) 5 mM ammonium formate with 0.01% formic acid, both containing 0.01% formic acid (Sigma Aldrich). The analysis was conducted at 40 °C, with a flow rate of 0.4 mL/min, an injection volume of 5 μL, and a total analysis time of 3 min. Mass spectrometer parameters included nitrogen as the source gas, a gas temperature of 300 °C, a gas flow rate of 9 L/min, and a heater temperature of 250 °C. Mass spectra were acquired in both positive and negative ionization modes at a spectral rate of 5.00 Hz, with ion source parameters set to 500 V end plate offset and capillary voltages of 4500 V (positive mode) and 3500 V (negative mode). The analysis was carried out on an LC-MS/MS system, consisting of an Agilent 1200 series liquid chromatograph coupled with an Agilent 6460 triple quadrupole system mass spectrometer with an electrospray ionization (ESI) source.

#### 2.4.2 Method Validation

The analytical method validation was carried out in accordance with the guidelines of the International Conference on Harmonization (ICH)^26^ Ensuring that the validation complies with national and international regulations, thereby increasing the reliability and applicability of the method. The validation parameters included selectivity, linearity, matrix effect, intraday and intermediate precision, and trueness. Selectivity was demonstrated by comparing the sample chromatogram with those of the diluent and standard solutions, and by differentiating between individuals of *P. torta*, *P. guianensis*, and *P. caimito*. Linearity and matrix effect were assessed by constructing three independent calibration curves using standards prepared in both the diluent and the plant matrix. Correlation coefficients and Pearson’s coefficients of determination were calculated. The matrix effect was evaluated by comparing the slopes of the calibration curves obtained from the plant matrix and the diluent using GraphPad Prism version 8.0. Intraday precision (repeatability) was assessed by injecting a standard preparation and six different samples in triplicate and single replicate, respectively. Intermediate precision was evaluated by having an external analyst perform the same experiment on a different day, and the recovery data from both days were compared using the Student’s *t* test. Trueness was determined at three levels (high, intermediate, and low) by spiking the samples with a standard solution and calculating the recovery rates. The limits of detection (LOD) and quantification (LOQ) were calculated based on the slope of the calibration curves obtained during the linearity assessment. The calculations followed the equations LOD = 3.3 × σ/*S* and LOQ = 10 × σ/*S*, where *S* represents the mean slope of the calibration curves, and σ corresponds to the standard deviation of the intercepts on the *y*-axis from the three linear calibration curves.

## 3 Results and Discussion

### 3.1 Optimization of the Extraction Conditions

The experimental design was developed following the principles of “Analytical Quality by Design” (AQbD),^27^ which included the optimization of analytical parameters. This strategy emphasizes a systematic and risk-based approach to method development. This framework allowed for the rational selection of critical variables based on prior knowledge and preliminary tests, contributing to the scientific basis of the optimization process. Together, these approaches underscore the methodological reliability and analytical quality of the study. The chromatogram and corresponding peaks, along with their ultraviolet profiles, are shown in Figure 1.

**Figure 1:**
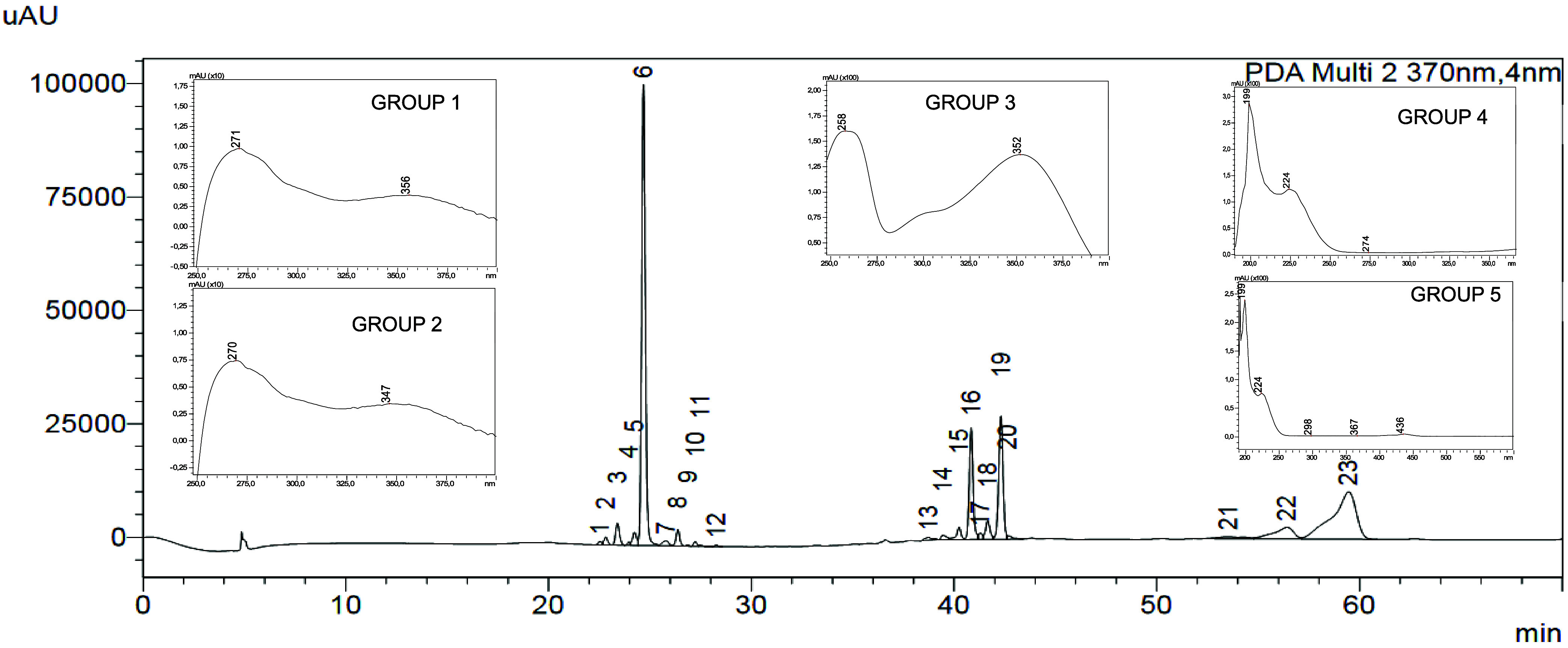
Majority peaks of HPLC-DAD chromatogram of extract of *P. guianensis* at 370nm with their grouped ultraviolet absorption spectra (See method item 2.4.1.). Group 1 (peaks 1, 2, 4, 5, 8, 11, and 12; λ_max_ = 271, 356 nm), Group 2 (peaks 3, 7, 9, 10; λ_max_ = 270, 347 nm), Group 3 (peak 6; λ_max_ = 258, 352 nm), Group 4 (peaks 13 to 20; λ_max_ = 224, 274 nm), and Group 5 (peaks 21 to 23; λ_max_ = 224, 436 nm).

Within the genus *Pouteria*, there is a broad range of phenolic compounds, particularly flavonoids, which exhibit pharmacological activities that correlate directly with their ethnopharmacological uses. These activities include well-documented antioxidant, anti-inflammatory, and immunomodulatory effects.^28,29^ Due to their therapeutic potential, flavonoids were selected as the group for optimizing extraction conditions from *P. guianensis* leaves. The peaks of interest in the chromatogram (1 to 12) were chosen based on the UV profile, presented in Figure 1.

To date, there have been no published studies assessing the impact of extraction conditions on *Pouteria* spp. leaves. Previous research has focused primarily on the fruits and seeds of the genus, evaluating factors such as sonication time, temperature, and the plant drug/solvent ratio, to estimate the variability ranges of these parameters.^22–24^ Based on methods reported in the literature for the genus and the promising extraction properties, including effective extraction and preservation of plant metabolites, we selected ultrasonic bath extraction for this study.^30^

Following the optimization tests and data analysis, the model and curvature were found to be statistically significant (*p* < 0.05). Linear regression analysis yielded an *R* ^2^ of 0.9846, an adjusted *R* ^2^ of 0.9702, and a coefficient of variation of 1.75%. The complete 2^4^ factorial design, which included two levels, a central point, and replication, demonstrated a predictive trueness of 99.9% for all analyzed variables.

Analysis of variance (ANOVA) revealed a strong correlation among all evaluated conditions, except for the plant drug/solvent ratio, which showed a *p*-value of 0.5649, as summarized in Table 2.

**Table 2:** Evaluation of the Significance of the Variance of the Variables Chosen for Extractive Optimization and Their Correlations

variable	sum of squares	*df*	mean of squares	*F*-value	*p*-value
model	2.50 × 10^16^	15	1.66 × 10^15^	68.39	<0.0001
A-sonication time	3.58 × 10^15^	1	3.58 × 10^15^	14.33	<0.0001
B- number of cycles	1.02 × 10^16^	1	1.02 × 10^16^	421.04	<0.0001
C-temperature	1.20 × 10^15^	1	1.20 × 10^15^	49.24	<0.0001
D- drug/solvent ratio	8.41 × 10^12^	1	8.41 × 10^12^	0.3455	0.5649
AB	1.35 × 10^15^	1	1.35 × 10^15^	55.60	<0.0001
AC	4.12 × 10^14^	1	4.12 × 10^14^	16.92	0.0008
AD	2.94 × 10^14^	1	2.94 × 10^14^	12.09	0.0031
BC	5.24 × 10^13^	1	5.24 × 10^13^	2.15	0.1618
BD	5.75 × 10^14^	1	5.75 × 10^14^	23.63	0.0002
CD	2.19 × 10^14^	1	2.19 × 10^14^	8.98	0.0085
ABC	1.52 × 10^15^	1	1.52 × 10^15^	62.40	<0.0001
ABD	2.86 × 10^15^	1	2.86 × 10^15^	117.40	<0.0001
ACD	4.89 × 10^14^	1	4.89 × 10^14^	20.08	0.0004
BCD	3.74 × 10^14^	1	3.74 × 10^14^	15.38	0.0012
ABCD	1.78 × 10^15^	1	1.78 × 10^15^	73.28	<0.0001
curvature	3.44 × 10^15^	1	3.44 × 10^15^	141.41	<0.0001
total error	3.89 × 10^14^	16	2.43 × 10^13^		
corrected error	2.88 × 10^16^	32			

Pareto analysis (Figure 2A) and tests for homoscedasticity (Figure 2B) indicated that the variables ABC, ACD, and temperature alone require careful control, as they significantly influence the total flavonoid content. Specifically, the negative correlation with temperature suggests that high temperatures may reduce the extraction efficiency. We hypothesize that the presence of the latex stored in laticiferous cells, a characteristic feature of the genus, may disrupt the organelle membrane at high temperatures, resulting in the trap of the metabolites within a viscous network.^13^

**Figure 2:**
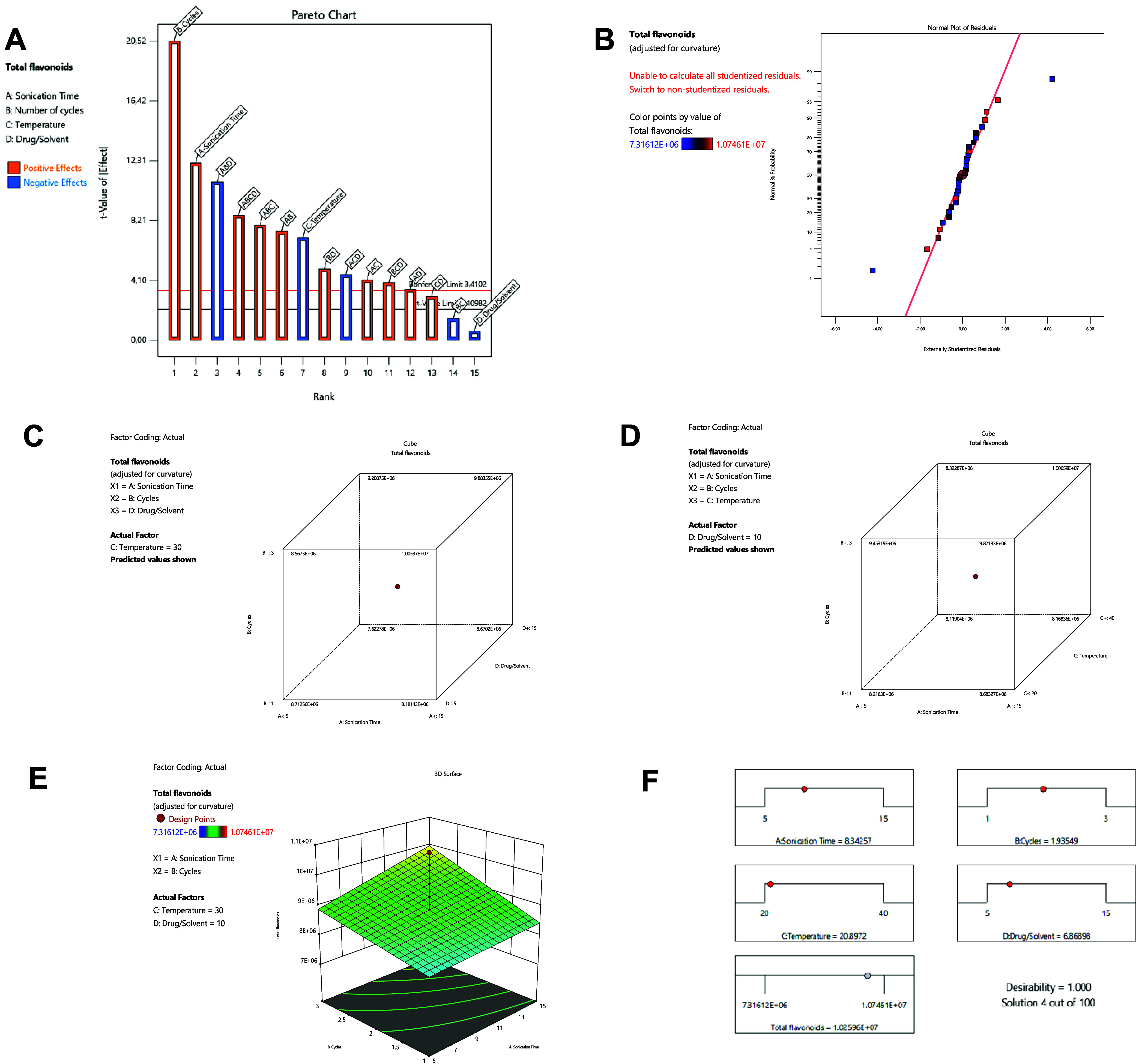
Pareto diagram (A), QQ-plot of the homoscedasticity of the residues (B); ternary graphs of the most influential correlations (C, D); surface graph of the most influential correlation in the model (E) and final extractive conditions.

The ternary and binary correlations with the greatest impact on the model are shown in Figure 2C–E, respectively. The data indicate that low drug/solvent ratios (level −1) do not adversely affect the extraction capacity if the other variables are set to their upper limits (+1). However, in the absence of an effect from the drug/solvent ratio, all other variables must be at their upper limits to optimize the extraction process. This suggests that the drug/solvent ratio may not act as a deterministic variable but rather plays a modulating role in the system.^21^

When all four variables are considered, the maximum extraction capacity of the metabolites approached the central value (level 0) for most parameters, except for the drug/solvent ratio, which remained intermediate between levels −1 and 0 (Figure 2F). The optimal extraction conditions identified were a low temperature (21 °C), an extraction time of 8 min, two extraction cycles, and a drug/solvent ratio of 7. This combination of factors not only optimizes the extraction process but also contributes to the efficient use of plant material, energy, and time. Furthermore, a reduction in costs is also expected, and these optimized conditions could be scaled up for commercial and industrial applications.^31^

Optimization within the *Pouteria* genus remains largely underexplored in the scientific literature, with only a limited number of studies applying statistical approaches to improve extraction or processing methodologies involving species from this group. One such study by Sathishkumar et al. (2011) employed a Taguchi L16 orthogonal array design to optimize the extraction of α-amylase inhibitors from *Pouteria sapota* seeds, investigating variables such as solvent type, extraction time, and temperature. Similarly, Soares et al. utilized a 2^3^ factorial design to optimize the formulation of jams produced from *Pouteria cf. gardneriana*, assessing the effects of different ingredient ratios on the product’s nutritional and functional properties. In another example, Tacias-Pascacio (2021) et al. applied a central composite design combined with response surface methodology (RSM) to enhance the aqueous enzymatic extraction of oil *from* *P. sapota* seeds, varying factors such as enzyme concentration, incubation time, and agitation speed. Thus, the present study represents the first to evaluate the impact of these variables on the leaves of the genus, providing a novel approach to the optimization of processes applied to this botanical matrix.

### 3.2 Characterization of the Analytical Marker

Recent reports have identified myricitrin as the majority peak in *Pouteria* spp, encouraging confirmatory analyses. Given the chemical similarities between myricitrin and other flavonoids, such as isoquercitrin, liquid chromatography coupled with high-resolution mass spectrometry (LC-HRMS) was employed for further investigation. The metabolite has been previously identified twice within the genus: in the leaves of *P. torta* ^32^ and in the seeds of *Pouteria lucuma*.^33^ Furthermore, its aglycone has been identified in the fruits of *P. macrophylla*,^34^ the seeds of *P. lucuma*,^33^ and the leaves of *Pouteria campechiana*.^35^

Two distinct fragmentation patterns, as referenced in MassBank,^25^ were investigated in both positive and negative ion modes. The total ion chromatogram and corresponding transition ions are presented in Figure 3. Fragmentation analysis revealed the loss of a rhamnose moiety (*m*/*z* 146), which corresponds to the sugar component of the myricetin aglycone, as well as the cleavage of the B-ring bond of the flavonoid skeleton (*m*/*z* 46).^36,37^

**Figure 3:**
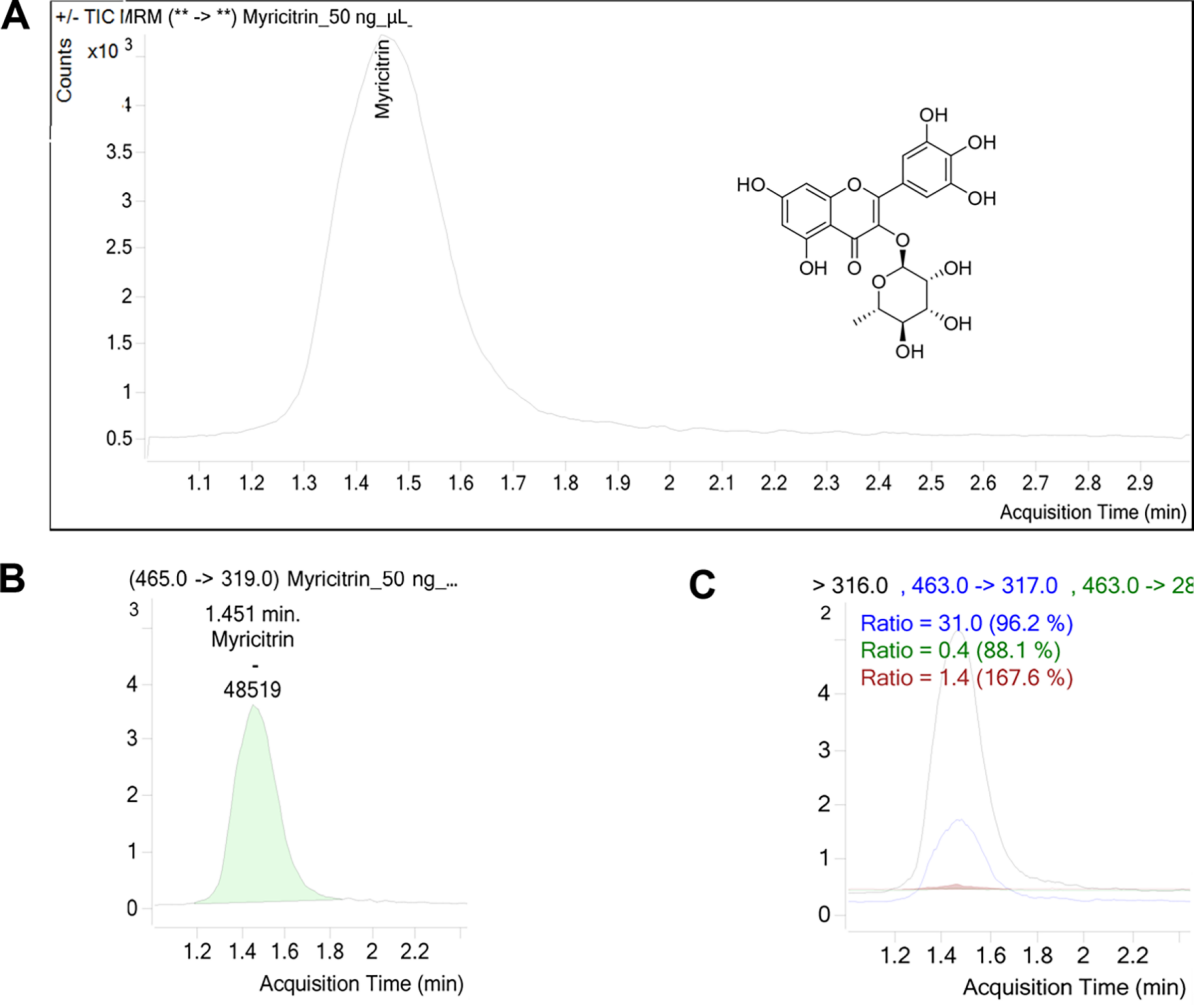
Chromatogram of total ions obtained from the direct injection of the 80% hydroethanolic extract of *P. guianensis* (Aubl.) on HPLC-QTRAP-MS/MS (A) and spectra in positive (B) and negative mode (C).

A myricitrin standard was coinjected with the sample, and the results confirmed that both eluted at identical retention times and exhibited matching ultraviolet spectra (Figure 4). This validation confirms that flavonoids are the predominant substances in this species.

**Figure 4:**
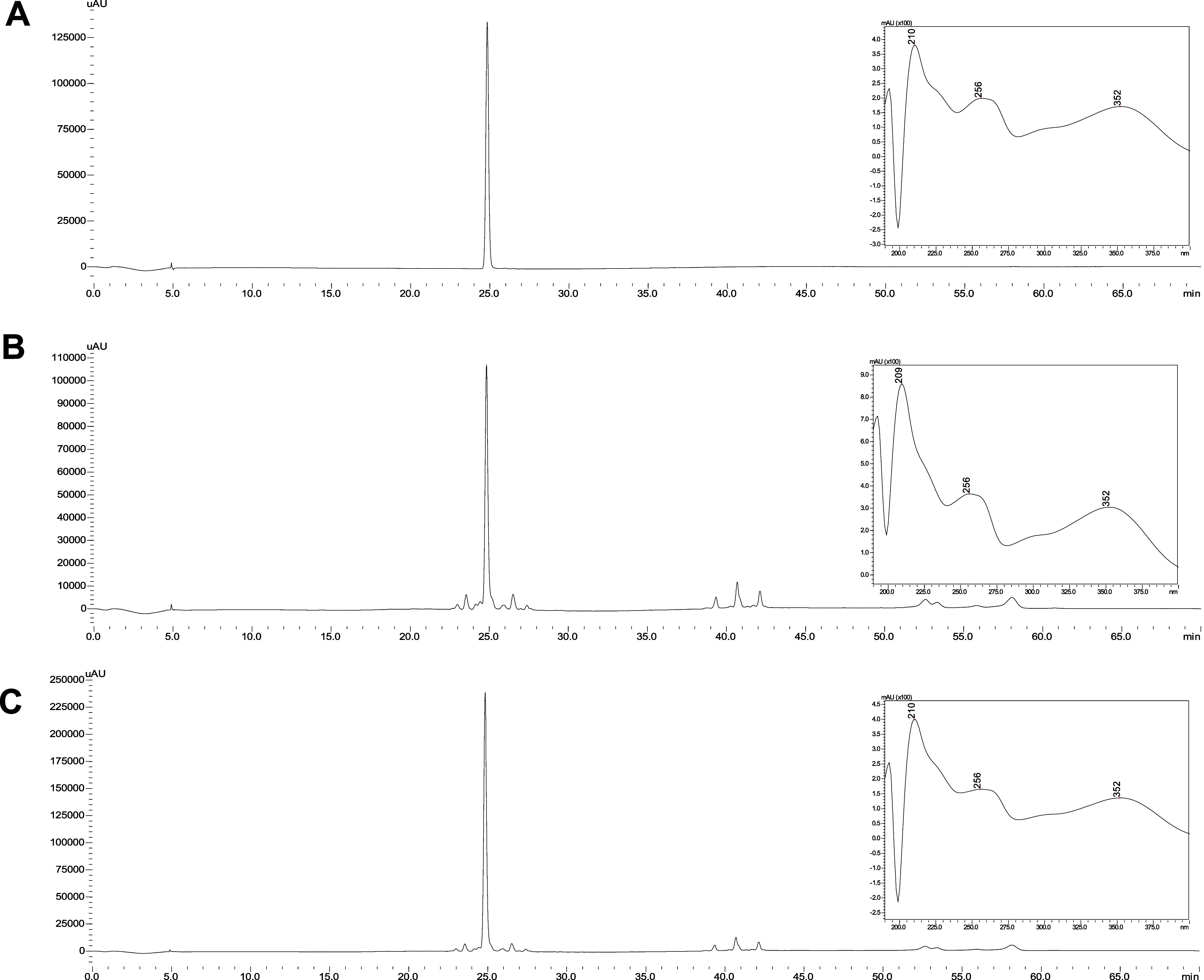
HPLC-DAD chromatograms: (A) myricitrin standard (65.8 μg/mL), (B) *P. guianensis* (Aubl.) sample (10 mg/mL) and (C) *P. guianensis* (Aubl.) sample contaminated with myricitrin standard and their respective spectra in the ultraviolet region.

### 3.3 Validation

#### 3.3.1 Selectivity

In this study, selectivity was evaluated in two different stages, as illustrated in Figure 5. In the first stage (Figure 5A), the selectivity was assessed using a method based on measurement errors.^38^ This approach demonstrated that the analyte exhibits a unique retention time, distinct from any peaks observed in the diluent, does not interfere with the standard, and is therefore confirmed as pure.

**Figure 5:**
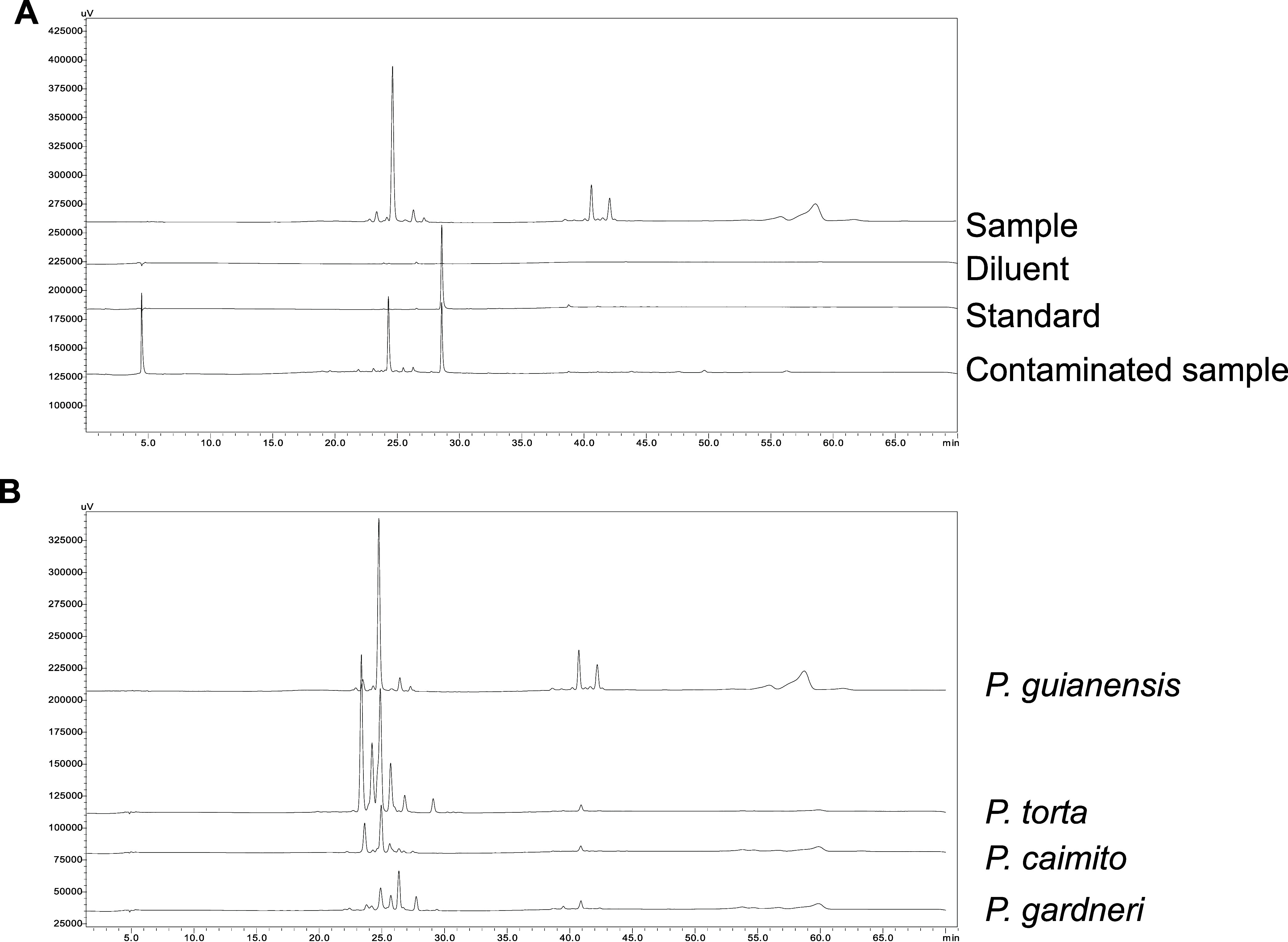
Selectivity profile of the analytical method, with comparison of analytical influences (A) and between different individuals of the *Pouteria* sp. genus (B).

The second stage focused on assessing the variability of plant chemodiversity in response to environmental factors, particularly climatic variations across different seasons.^39–41^

Finally, differentiation among species within the genus was carried out, as shown in Figure 5B. As previously noted, species within the same plant genus, despite showing metabolic variability, often share similar compound profiles.^42,43^ Similarly, while species within the *Pouteria* genus display variation in chemical composition, phenolic compounds especially flavonoids, are commonly reported.^9,44–46^ However, although there are qualitative and quantitative differences in the chemical profiles of these species, these variations enable the use of the method as an auxiliary tool for botanical differentiation.

#### 3.3.2 Linearity and Matrix Effect

The property of linearity reflects the ability of the method to yield results that are directly proportional to the concentration of the analyte.^47^ However, when using a quantification method with standard preparation in a diluent, it is essential to verify that matrix components do not interfere with the quantification of the standard, either by reducing or enhancing its measured concentration.^48^ The results of the three analytical curves for the standard, both in the diluent and the plant matrix, are shown in Table 3 at concentrations of 14.7, 22.1, 29.5, 36.9, and 44.2 μg/mL of quercetin, along with the findings from the matrix effect assessment and the calibration curve preparation in the plant matrix.

**Table 3:** Statistical Evaluations of Parametricity Requirements, Variance and Correlation of the Slope of the Matrix Effect Curves and Linearity

tests	linearity	matrix effect	parameter	result
Cochran test	*C*_tab_ = 0.684	*C*_tab_ = 0.684	*C*_calc_ < *C*_tab_	homoscedastic
	*C*_calc_ = 0.447	*C*_calc_ = 0.496		
Pearson’s correlation coefficient (*R*)	0.999	0.998	*R* > 0.990	highly correlated variables
coefficient of determination (*R* ^*2*^)	0.999	0.996	*R*^*2*^ > 0.980	linear association
Snedecor’s *F*-test (for variance)	*F*_tab_ = 4.67	*F*_tab_ = 4.67	*F*_calc_ ≥ *F*_(5%,1,*n*‑2)_, *p* > 0.05	similar variances
	*F*_calc_ = 4.89	*F*_calc_ = 11.94	It is indicative of which *T*-test methodology should be carried out and not normative	
	*p* > 0.0001	*p* > 0.0001		
Shapiro–Wilk	*p* > 0.05	*p* > 0.05	*p* > 0.05	homoscedastic
Student’s *t* test (assessment of whether the angular coefficient ≠ 0)	*p* < 0.0001	*p* < 0.0001	*p* < 0.05	coefficient ≠ 0
comparison of intercepts of curve in diluent and curve in matrix	*F*_(5.23)_ = 0.3957; *p* = 0.8466		*p* > 0.05	coefficients with no statistical difference

Following the evaluation of these parameters, it was confirmed that no matrix effect was present, allowing accurate quantification in the analytical method for samples containing the standard. This approach is preferable to preparing the standard in a diluent, as it simplifies sample preparation and conserves both time and reagents during analytical runs.

Additionally, calibration curves were generated for myricitrin (*y* = −43293.5 + 27587986.8*x*) and quercetin (*y* = 254069.4 + 66269367.91*x*) in the solvent, at the same concentrations evaluated in the linearity study (14.7, 22.1, 29.5, 36.9, and 44.2 μg/mL), with three standards prepared for each concentration level. The ratio of the slope coefficients of these curves was found to be 2.40, indicating that by analyzing the *P. guianensis* samples and dividing the results by this factor, the actual concentration of myricitrin can be determined. This approach allows for the adjustment of the substance’s response factor.

#### 3.3.3 Limits of Detection (LOD) and Quantification (LOQ)

Three independent calibration curves were constructed within the previously established linear range (*y* = 66,509,217.3*x* – 42,596.8; *y* = 67,109,292.6*x* – 93,034.4; *y* = 65,922,903.2*x* – 52,810.2), allowing for the determination of the limits of detection (LOD) and quantification (LOQ) based on the slope (S) and the standard deviation of the *y*-intercepts (σ). The calculated LOD and LOQ were 0.00132 and 0.00401 μg/mL, respectively. These values demonstrate the high sensitivity of the method and its adequacy for the quantitative analysis of the target analyte.

#### 3.3.4 Precision and Intermediate Precision

The precision of the method was assessed through intraday repeatability and intermediate precision, which are essential for evaluating its reproducibility and ensuring the reliability of the analytical conditions. These parameters are influenced by factors such as calibrated materials and controlled environmental conditions.^49^ The results of this evaluation are summarized in Table 4.

**Table 4:** Evaluation of the Repeatability (Analyst 1, Day 1) and Reproducibility (Analyst 2, Day 2) of Myricitrin Assay As a Function of Quercetin^a^

analyst 1			analyst 2			reproducibility		
sample content (μg/mL)	recovery (%)	RSD (%)	sample content (μg/mL)	recovery (%)	RSD (%)	intermediary precision RSD (%)	*t* test	*p* value
0.0303	102.4	1.3	0.0306	103.5	1.3	1.2	*T*_calculated_ = 0.11; *T*_tabulated_ = 1.81; *df* = 10	0.7392
0.0325	104.9		0.0307	104				
0.0297	100.7		0.0304	103				
0.0303	102.8		0.0302	102.4				
0.0303	102.5		0.0296	100.3				
0.0324	103.2		0.0300	101.7				

a Legend: RSD = Relative Standard Deviation.

Considering the diluted sample concentration of 29.53 μg/mL of myricitrin relative to quercetin, the concentration of myricitrin in the plant was calculated to be 2.96 mg/g (0.30%). According to ICH guidelines,^26^ the acceptable limits for repeatability and reproducibility are 3.7 and 6%, respectively. These results confirm the trueness of the method and suggest no significant analyst variability under the specified instrumental conditions.

#### 3.3.5 Trueness

Trueness reflects the ability of the method to yield true values in analytical measurements, reducing α-type errors in chemical analysis.^50^ To evaluate trueness, samples were spiked with 100% standard at three different concentration levels, and the results are presented in Table 5.

**Table 5:** Recovery of the Analyte to Assess the Trueness of the Method at Low, Intermediate, and High Levels

level (%)	recovery (%)	average (%)	RSD (%)
50	98.53	98.40	0.20
	98.18		
	98.49		
100	102.66	100.66	1.73
	99.64		
	99.67		
150	97.82	97.57	0.29
	97.63		
	97.26		

According to ICH guidelines,^26^ the expected recovery for samples with a 0.30% concentration should range from 95 to 105%, as shown in Table 5. In addition, the variability among replicate measurements remained within the repeatability limits, confirming the trueness of the method and alignment with the proposed analytical strategies.

### 3.4 Hydrolysis of Samples of the Genus *Pouteria* sp

Due to the botanical similarity within the *Pouteria* genus, one effective approach for species differentiation is the hydrolysis of labile compounds, which facilitates identification based on chemical markers.^51,52^ Usually, different species contain compounds with similar phenolic cores but vary in the sugar moiety positions, allowing for the identification of distinct aglycones in plant extracts.^53^ Moreover, the sugars attached to these compounds can influence the polarity of the core structure, potentially causing coelution during HPLC-DAD analysis and leading to the loss of species-specific information.^54–56^

The results of hydrolysis for the four *Pouteria* species analyzed in this study are shown in Figure 6. Notable changes in the qualitative chromatographic profile were observed, particularly in the flavonoid region, where a significant reduction in the number of chromatographic peaks was evident. Specifically, the analysis indicated that the *P. guianensis* and *P. torta* share the same aglycones, although differences in the attached sugars may exist.

**Figure 6:**
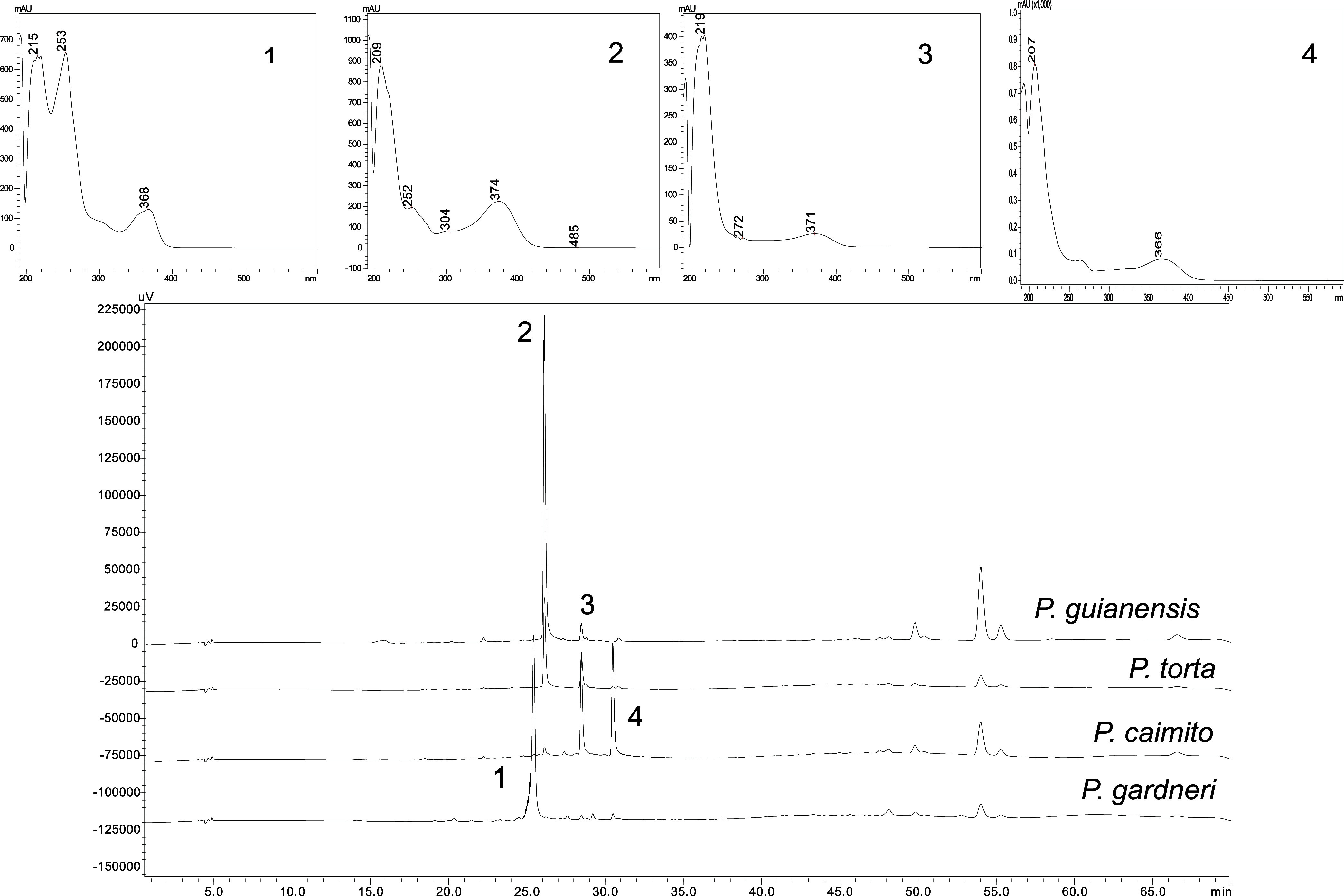
Chromatograms and UV profiles of hydrolysis profile of samples from *Pouteria* sp. species (Peak 1 λ_max_ = 215, 253,368; Peak 2 λ_max_ = 209,252,374; Peak 3 λ_max_ = 218, 371 and Peak 4 λ_max_ = 207,366).

In addition, a unique aglycone was identified in *P. caimito* that was absent in the other species, highlighting the potential for distinguishing this species from others, despite the high botanical and genetic similarity between *P. caimito* and *P. guianensis*.^12^ This finding underscores the value of using various methods and tools for accurately differentiating these species.

The ultraviolet spectrum of the *P. guianensis* sample revealed a peak with an absorption band consistent with myricetin, the aglycone of myricitrin.^46^ Furthermore, peak 1 exhibited ultraviolet absorption characteristics similar to those of quercetin derivatives, suggesting the presence of quercetin or its derivatives in the genus and validating the use of this compound as an external marker for identification.^57^

## 4 Conclusions

This study represents the first pharmacognostic characterization and optimization of flavonoid extraction from *P. guianensis* leaves, achieved under conditions of 21 °C, 8 min of sonication, two cycles, and a drug/solvent ratio of 7. These findings emphasize the critical role of temperature control in optimizing the extraction process. A selective method was developed and validated for differentiating species within the *Pouteria* genus, focusing on both glycoside and aglycone compounds through acid hydrolysis. The method validation identified myricitrin as a marker compound, with quercetin used as an external standard. Although quercetin is reported in the genus, it was not detected in any of the species evaluated in this study. Moving forward, scaling up the extraction conditions to industrial levels should be explored, along with a pharmacological evaluation of the ethnopharmacological properties of the optimized extract.

## Abbreviations

ANOVA: analysis of variance
AQbD: analytical quality by design
DAD: diode array detector
DPPH: 2-diphenyl-1-picrylhydrazyl
HPLC: high performance liquid chromatography
ICH: international conference on harmonization
LC-HRMS: liquid chromatography coupled with high-resolution mass spectrometry
Min: minutes
RSD: relative standard deviation
UV: ultraviolet
